# City Hospital

**Published:** 1880-09

**Authors:** 


### EDITORIAL DEPARTMENT.

"If thou hast truth to utter, speak;
And leave the rest to God."

City Hospital.—We promised in a late issue, to advert to this matter again, and will do so now as concisely as may be consistent with the importance of the subject. As stated heretofore, the caption of this paragraph is a misnomer when applied to the building at the northwest corner of Calvert and Saratoga streets. This house was known some years ago, as Public Grammar School, No. 9: late, as Washington University, and now as the College of Physicians and Surgeons. The location and building are unsuited as a hospital for the proper professional treatment of individuals of *any race, nationality, sex, or condition* of mankind. Its proximity to anatomical and surgical lecture rooms would alone render it unfit for the proper accomodation of *any class* of patients, as they should be cared for, and especially so, for the indigent, and un-informed, who must in the nature of things find asylum, more or less often, in an institution bearing the name "City Hospital," and receiving the patronage and support of the municipal authorities! An intelligent stranger though he should hail from New Zealand, in seeking for a "City Hospital," would hardly persue his way to the N. W. corner of Saratoga and Calvert Streets; but should his footsteps be diverted thither by one un-acquainted with the building bearing, this inappropriate appellation! the in-eligible spot, and unfavorable hygienic surroundings, would cause him very rationally to conclude, that however much our city had fallen into the current of enlightened and progressive improvement, in *other* important particulars; she had relegated the laws of health to the science of the middle ages, when adopting such an establishment for her sick and suffering poor. The building is ill-suited, per se, for *any kind of hospital*, so much so, indeed, that it is not probable Mr. Bergh would willingly receive it for the treatment of inferior animals is situated in a low damp spot, at the foot of a high hill, all the waters, and debris, from which during a shower, or thaw of snow, are emptied into a large sewer at its outer corner. It would be folly to suppose that the gaseous emanations from this source alone, to say nothing of those from the surrounding marshy, and damp soil, are not deleterious to the health even of the well, who are compelled to inhale them for any considerable period of time: and if so, must not such effluvia exert a still more pernicious influence upon the unfortunate victims of accident, or disease, who are *compelled* to seek the asylum thus arranged for them by authority, and enter the portals of this building for medical or surgical treatment? But these exhalations are only among several other serious objections to its use as a hospital for human beings, potential, and obvious as they are even to the casual observer. The house is situated immediately on a corner, where two streets cross at right angles, and where it is probable as many vehicles of a heavy character, and street cars with jingling bells pass, and repass over one of them, (Calvert,) within twenty-four hours, as that of any other thoroughfare, except Baltimore street, within the limits of the entire City of Baltimore! But seemingly as if to "add insult to injury," the indigent and unfortunate classes, who are forced to take shelter in this house, in their dire necessity, if not conscious of it or admission may soon be made aware of the fact, that in another part of the building, dissections of the dead human body were almost constantly practiced by medical students! Comment upon such a state of things would seem a work of supererogation, as certainly the simple statement, of facts already made, in regard to *this* "City Hospital," ought to be sufficient to cause an abatement of it as soon as practicable. This establishment seems to be conducted solely in *the interest* of ten or a dozen medical men, *probably, in no wise superior to any similar number*, of "the rank and file" of "the gallant six hundred," whose names appear on the list of "Physicians of Baltimore!" But want of space admonishes that we must defer additional remarks to another occasion.

TROMMER'S EXTRACT OF MALT.—Our acquaintance with this valuable preparation began several years ago, and we were among the first of the professsion to prescribe it frequently in Baltimore.

Time has tended only to strengthen the favorable opinion we expressed in a letter to the manufacturers of Trommer's Extract of Malt, a few years ago. Of the conditions of the system in which it may be used with profit, it is unnecessary for us to speak editorially, as the professsion is aware of the great value of malt in the successful treatment of debility, and many forms of actual disease.

LACTOPEPTINE.—We have no hesitation in calling attention to this excellent adjuvant in the treatment of the gastro-intestinal troubles, that are prostrating so large a number of the infant population, and hastening many of them to their early graves, during the present hot weather, in our cities and towns. Experience with this article during the present "heated term," in the treatment of the so-called "summer complaints," of infants and children, enables us to speak of it in high terms of commendation, and as the most valuable auxiliary to the remedies we have prescribed for this class of sufferers, at any time.

A sample of Lactopeptine was sent us from New York, by the

